# Family management of common sleep disturbances among children with autism: Implications for pediatric nursing research and practice

**DOI:** 10.1016/j.pedn.2025.03.006

**Published:** 2025-03-15

**Authors:** Shayleigh Dickson Page, Lindsey Clark, Margaret C. Souders, Jennifer A. Pinto-Martin, Janet A. Deatrick

**Affiliations:** a University of Pennsylvania School of Nursing, 418 Curie Blvd, Philadelphia, PA 19104, United States of America; b University of Pennsylvania Perelman School of Medicine, 3400 Civic Center Blvd, Philadelphia, PA 19104, United States of America; c Children’s Hospital of Philadelphia, 3401 Civic Center Blvd, Philadelphia, PA 19104, United States of America

**Keywords:** Sleep, Family management, Autism spectrum disorder

## Abstract

**Background:**

Sleep disturbances are common among children with autism spectrum disorder (ASD) and can negatively impact the health and wellbeing of the child, caregiver, and family. Nurses are well-positioned to support families of children with ASD to improve sleep.

**Design & purpose:**

In this mixed methods study, we leveraged an existing dataset to 1) characterize qualitative descriptions of sleep disturbances experienced by children (4–10y) with ASD, 2) examine the convergence of qualitative descriptions of sleep disturbances with quantitative scores on the Children’s Sleep Habits Questionnaire (CSHQ), and 3) explore strategies used to manage bedtime and sleep disturbances.

**Results:**

In this sample (*n* = 30), 70 % of caregivers described that their child had one or more sleep disturbances, with night wakings (43.3 %), bedtime resistance (30 %), and sleep anxiety (30 %) being most common. Qualitative descriptions largely converged with the CSHQ scores; however, in 20 % of cases, the caregiver reported no concerns about sleep while the CSHQ score indicated a clinically significant sleep problem. Management of bedtime and sleep disturbances required significant effort and balancing of multiple domains, including the child’s sleep needs, the sleep needs of the caregiver and other family members, the child’s sleep environment preferences and daytime activities that promote or disrupt sleep.

**Conclusion & implications to practice:**

Sleep disturbances are prevalent, despite efforts to implement bedtime routines and manage sleep disturbances. Pediatric nurses play an integral role in screening for sleep disturbances, educating families, and providing guidance for implementing behavioral and environmental interventions. Implications for clinical practice and future research are discussed.

Children with autism spectrum disorder (ASD) often experience sleep disturbances. ASD is a neurodevelopmental condition characterized by differences in social communication and patterns of behavior that are restricted and repetitive (American Psychiatric Association, 2013). A large body of literature documents that sleep disturbances are associated with greater severity of core ASD behaviors (i.e., social differences, repetitive behaviors) and increased daytime behavioral problems, such as irritability, aggression, and hyperactivity (Goldman et al., 2011; Hollway et al., 2013; Johnson et al., 2018; Malow et al., 2006; Mazurek & Sohl, 2016).

Sleep disturbances encompass an array of challenges related to sleep, including insufficient sleep quantity for age, poor sleep quality, inappropriate timing of the sleep period, and excessive daytime sleepiness (Mindell & Owens, 2015). The prevalence of sleep disturbances among children with ASD ranges from 50 to 80 %, with variability due to sample characteristics and method of sleep measurement (Goldman et al., 2012; Johnson et al., 2012; Krakowiak et al., 2008; Malow et al., 2016; Souders et al., 2009). By comparison, about 20–40 % of children with typical development have sleep disturbances (Calhoun et al., 2014; Fricke-Oerkermann et al., 2007; Owens, Spirito, & McGuinn, 2000). In ASD, sleep disturbances often emerge in infant and toddler years (Humphreys et al., 2014; MacDuffie et al., 2020; Reynolds et al., 2019) and persist into adulthood (Charlton et al., 2023; Jovevska et al., 2020). While sleep disturbances among children with typical development may remit or become self-managed as the child develops, prior qualitative work suggests that the persistent and unrelenting nature of sleep disturbances in ASD can lead to feelings of overwhelm and hopelessness for caregivers (Halliwell et al., 2021).

Sleep disturbances have a multitude of impacts on the child, caregiver, and family. Distruptive daytime behaviors as well as daytime sleepiness may negatively impact the child’s engagement in school and in therapy services, therby affecting learning and developmental progress. Sleep disturbances also impact cardiovascular and metabolic health in the general pediatric and adult populations (Cappuccio et al., 2008; Lian et al., 2019). Recent research among adults with ASD high-lighted that poorer sleep quality is associated with having bodyweight in the overweight or obesity range and increased risk factors for cardiovascular disease (Bishop et al., 2023). As expected, parental sleep quality is also negatively impacted when the child experiences disrupted sleep (Meltzer, 2008; Micsinszki et al., 2018; Micsinszki et al., 2023; Nic Ghiolla Phadraig & Smyth, 2023). Further, child sleep disturbances are associated with parents experiencing increased stress and higher levels of anxiety and depression (Johnson et al., 2018; Mannion & Leader, 2023; Martin et al., 2019). At the family level, family functioning and family resilience are lower when the child with ASD has sleep disturbances (Nic Ghiolla Phadraig & Smyth, 2023; Roberts et al., 2017). A qualitative study using focus groups further identified that the child with ASD having insomnia contributes to feelings of stress and anxiety across family members and results in significant emotional, social, economic, and educational consequences for families (Kirkpatrick et al., 2019).

Given the high prevalence and pervasive impacts of sleep disturbances on children with ASD and their families, early identification and treatment of sleep disturbances is of high importance to providing clinical care. Clinical guidelines recommend screening all children with ASD at least annually for sleep disturbances, evaluating for and managing contributing medical factors (e.g., constipation, anxiety, sleep-disordered breathing), beginning therapeutic intervention with behavioral approaches, and using pharmacologic therapy (e.g., melatonin) when behavioral-intervention only approaches are not feasible or effective (Galion et al., 2023; Malow et al., 2012). Following implementation of these recommendations, clinicians must also ensure timely follow-up to evaluate for resolution of the sleep disturbance and escalation to a sleep specialist, if the sleep disturbance persists. Unfortunately, constraints on primary care visit time, provider knowledge about sleep and sleep disorders, and limited availability of sleep specialists can be barriers to implementing the guidelines (Galion et al., 2023; Malow et al., 2012). Notable gaps also exist in understanding the implementation of these guidelines from a family perspective.

Pediatric nurses are uniquely positioned to support efforts to screen for and treat sleep disturbances among children with ASD as they interact with families across primary care, school, community, and hospital settings. While the evidence-base for the effectiveness of various sleep interventions is growing, there are gaps in understanding how to implement sleep interventions across the spectrum of presentations of ASD and within diverse family environments. A recent scoping review of behavioral sleep interventions across pediatric populations found that behavioral sleep interventions for insomnia are generally effective; however, in implementing these interventions clinicians may face challenges as they must evaluate family strengths, identify barriers, and then select from an array of recommended intervention components (Meltzer et al., 2021). Specific to children with ASD, a meta-synthesis of pharmacologic (e.g., melatonin) and non-pharmacologic sleep interventions concluded that there is evidence to support the effectiveness of melatonin, behavioral interventions, and parent education programs for treating sleep disturbances (Cuomo et al., 2017). The authors further identify that there are critical gaps in our understanding of how to combine therapeutic approaches and limited studies have evaluated the efficacy of interventions within subgroups of children with ASD who experience specific sleep disturbances. This research is needed to support clinical decision-making and understanding what sleep guidance is needed in what circumstances.

To begin to address the knowledge gaps related to implementation of sleep interventions for children with ASD, we used the Family Management Style Framework (FMSF) as the theoretical framework to explore how caregivers experience and manage their child’s sleep disturbances. The FMSF outlines the family’s understanding of the child’s condition(s) and treatment regimen, the management behaviors used, and the perceived consequences of the child’s condition(s) for the child and family (Knafl et al., 2012). Applying this framework to the management of sleep disturbances, we can gain a deeper understanding of how caregivers view the child’s sleep disturbances and the specific strategies they use to manage sleep disturbances and daytime consequences. These findings can in turn be used to make recommendations for how pediatric nurses can best support families in implementing sleep interventions.

## Purpose

The specific aims of this mixed methods study are to 1) characterize qualitative descriptions of common sleep disturbances experienced by children (4–10y) with ASD and their families, 2) examine the convergence of qualitative descriptions of sleep disturbances with quantitative scores on the Children’s Sleep Habits Questionnaire (CSHQ), and 3) explore the strategies that caregivers use to manage bedtime and sleep disturbances.

### Design

We conducted a secondary analysis of data collected for a parent study on family management of caregiving challenges among families of children with ASD or Down syndrome. In this paper, we report only on data from caregivers of children with ASD because they completed in-depth interviews in addition to quantitative measures for the parent study.

### Parent study

Details of the parent study are described elsewhere (Page, Souders, Aryal, Pinto-Martin, & Deatrick, 2024; Page et al., in press). Briefly, primary caregivers of children (4–10y) with ASD and primary caregivers of children with Down syndrome were recruited from an urban children’s hospital in the northeast using e-mail outreach via the electronic medical record, posting on hospital-affiliated social media pages and newsletters, and flyers displayed in pediatric outpatient clinics. We first compared the six dimensions of family management, quantitatively measured by the Family Management Measure (FaMM), between families of children with ASD and families of children with Down syndrome (Page, Souders, Aryal, Pinto-Martin, & Deatrick, 2024). Second, among caregivers of children with ASD, we compared and contrasted quantitatively defined family management patterns with qualitative descriptions of how caregivers manage daily caregiving challenges (Page et al., in press). Specific sleep disturbances were not systematically identified during the primary analysis and interpretation of the data.

## Methods

### Data Collection.

Sleep habits were assessed both quantitatively and qualitatively using the Children’s Sleep Habits Questionnaire (CSHQ) and interview transcripts from 30 primary caregivers of children with ASD. The CSHQ assesses sleep across eight subscales: bedtime resistance, sleep onset delay, sleep duration, sleep anxiety, night wakings, parasomnias, sleep-disordered breathing, and daytime sleepiness (Owens, Spirito, & McGuinn, 2000). Caregiver respondents recall “typical” sleep behaviors during a recent week and respond on a 3-point scale if the behavior “usually” (5–7 times/week), “sometimes” (2–4 times/week), or “rarely” (0–1 times/week) occurs. The total sleep problems score was the sum of all items, with higher scores indicating that sleep disturbances were more frequent. A total cut-off score of 41 (range 33–99) indicated children with clinically significant sleep disturbances (Owens, Spirito, & McGuinn, 2000). Internal consistency was good (Cronbach’s alpha = 0.874) in our sample.

Qualitative interviews were conducted by the first author within two weeks of completing the CSHQ. We used a semi-structured interview guide that was theoretically based on the FMSF and included specific questions about the management of bedtime routines and sleep challenges. For example, ‘What happens at bedtime at your home?’ and ‘Tell me about a recent bedtime or night that was challenging.’ Open-ended questions and probes (e.g., “tell me more about that” or “please give me an example of…”) were used to encourage participants to respond in detail. Interviews were conducted via Zoom, audio-recorded, and transcribed verbatim by either the first author (nine interviews) or a professional transcription service (21 interviews). The written transcripts were then compared to the original audio files by the first author to ensure the accuracy of transcription and complete de-identification. ATLAS.ti (Version 22) was used to organize and code the interview data.

### Data Analysis.

**Fig.1.**
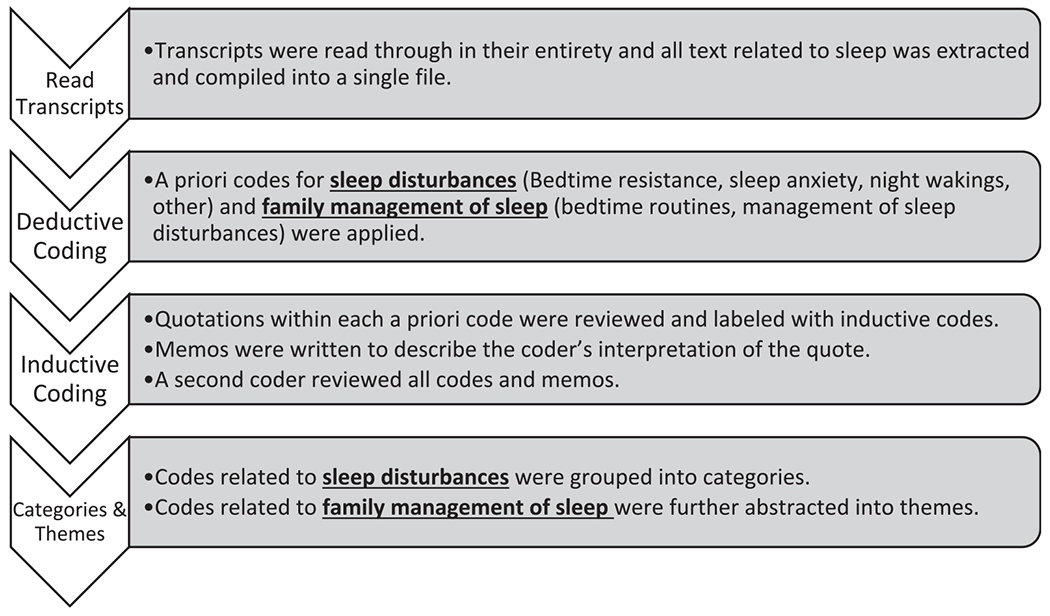
A Flow chart of Data Analysis Steps. This figure displays the steps of data analysis: (1) reading transcripts, (2) deductive coding, (3) inductive coding, (4) development of categories & themes.

From the interview data, we used directed content analysis (Fig. 1) to elicit (1) the types of sleep disturbances that children experience and (2) the strategies caregivers used to manage bedtime and caregiving challenges related to their child’s sleep disturbances (Graneheim & Lundman, 2004; Hsieh & Shannon, 2005). During an initial read of the interview transcripts, all passages related to sleep were extracted and compiled into a single file. A set of a priori codes was applied to the data to categorize content related to sleep disturbances (bedtime resistance, sleep anxiety, night wakings, other), and family management of sleep (bedtime routines, management of sleep disturbances). Next, the text within each category was iteratively reviewed and quotations were labeled with inductive codes and memos that described the underlying meaning (Graneheim & Lundman, 2004). Coding and memoing were completed by the first author and reviewed by the second author to confirm agreement with interpretations. Categories were used to group codes related to specific sleep disturbances and these were aligned with subscales of the CSHQ. Themes related to types of sleep disturbances were not created because of the concretized nature of the data. However, codes related to family management of sleep were further abstracted into categories then an overarching theme and three subthemes.

To quantify the types of sleep disturbances, an informational matrix was developed. Each row was a case (caregiver), and columns included quotations for each of the codes related to sleep disturbances. The first and second author, blinded to each other’s ratings, then reviewed the matrix to determine if sleep disturbances were present or absent in each case. Ratings were compared, and there was agreement in 29/30 cases, with the single discrepancy resolved via discussion.

To compare the quantitatively assessed CSHQ to the qualitative interview data, CSHQ scores were added to the informational matrix. The CSHQ score, using a cut-off of 41 to define sleep disturbances, was compared to the qualitative description of the sleep disturbances. Cases were labeled as convergent (CSHQ score and qualitative determination matched) or divergent (CSHQ score and qualitative determination did not match).

### Ethical considerations

The parent study received ethical approval from university (851299) and hospital (22–020150) institutional review boards. Data was collected from September 2022 to March 2023. Written informed consent was obtained from all participants, and verbal re-consent was obtained for participants that completed an interview. Consent included that data would be de-identified, stored for (7) years, and could be shared in the de-identified format for future research. The de-identified dataset was analyzed for this study.

## Results

### Sample characteristics

The sample was comprised of 30 primary caregivers (Table 1). The majority (83.3 %) were the child’s biological mother with three biological fathers, one adoptive mother, and one grandmother participating. Caregivers had a mean age of 38.67 ± 7.06. Nearly all (90 %) lived with a partner and the majority reported financial stability (75.9 %). Two-thirds of the children were male with a mean age of 6.90 ± 1.69.

### Sleep disturbances

Overall, 70 % of caregivers described that their child had at least one challenge related to bedtime or sleep. Primary sleep disturbances described by caregivers were night wakings, bedtime resistance, and sleep anxiety (Table 2).

Night wakings, which included any awakening after sleep onset but before waking for the day, were the most frequently described sleep disturbance and were reported by 43.3 % of caregivers. However, there was variability in how disruptive these night wakings were to caregivers or other family members. Some children had ‘independent night wakings’ in which the child awoke, occupied themselves (e.g., playing in their room), and returned to sleep without waking their caregiver. Other caregivers described that their child waking up was disruptive to their own sleep, and they needed tobe involved with helping the child return to sleep (see Managing Night Wakings). Bedtime resistance, reported by 30 % of caregivers, refers to difficulty settling down to sleep or attempting to delay bedtime by making requests or coming out of the bedroom after the caregiver had concluded the bedtime routine. Caregivers described that their child did not appear tired at bedtime. The amount of time it took for these children to finally fall asleep was variable, but for some extended to several hours. Sleep anxiety was described by 30 % of caregivers and manifested as the child needing the caregiver present to fall asleep, the child needing the caregiver present in order to maintain sleep through the night, and the child expressing specific fears about bedtime. Parasomnias were difficult to discern from caregiver descriptions of sleep and were not specifically probed during the interviews, thus these sleep difficulties were not quantitized. Caregiver descriptions of possible parasomnias included descriptions of tossing and turning or moving around the bed, nightmares, and/or night terrors. Reports of daytime sleepiness, early morning rising, and sleep disordered breathing (i.e. obstructive sleep apnea) were infrequent in this sample.

Qualitative descriptions of sleep disturbances converged with CSHQ scores in 80 % of cases. In 21 cases, the interview text described at least one sleep disturbance and the CSHQ score was above the cut-off of41 to indicate a clinically significant sleep concern. There were 3 cases in which the interview text described no sleep disturbances and the CSHQ was also below the cut off, thus these cases also converged. In 20 % of cases there was divergence between the qualitative description of sleep disturbances and the CSHQ score. In all cases, the caregiver reported their child had no sleep disturbances during the qualitative interview, but the CSHQ score indicated a clinically significant sleep concern, with scores ranging from 42 to 48.

### Family management of sleep

Across interviews and independent of the type of sleep disturbances, an overarching theme of ‘*Family Management of Sleep is A Balancing Act*’ was described. Caregivers balanced multiple domains to support their child’s sleep. Domains included the child’s sleep needs, the sleep needs of the caregiver and other family members, the child’s sleep environment preferences (including sensory needs), and daytime activities that promote or disrupt sleep. Subthemes of ‘Establishing Routines’, ‘Managing Night Wakings’, and ‘Management Effort’ are described in detail below and outline the specific areas in which caregivers engaged in this ‘balancing act’ (Table 3).

### Establishing routines

The majority of caregivers (86.7 %) endorsed having a consistent and predictable bedtime routine that they had established over time. Among bedtime routines, there was variability in caregiver involvement and the rigidity of the routine. When caregiver involvement in the bedtime routine was high, there was increased effort devoted to helping the child through the steps of the routine. In contrast, other caregivers reported their child led the routine rather independently. The use of a visual schedule was one strategy to promote independence with the bedtime routine. For example, one mother showed on video a visual schedule and explained, “*I have, like a whole schedule up, like it’s actually one over there, like on the wall, so they know, like what they have to do every night” (Participant 3)*. Rigidity of the routine also varied. Some caregivers described specific steps that were necessary to carry out in the same way each night, while others endorsed a more flexible set of activities before bedtime, such as brushing teeth, reading a story, or singing a song. Caregivers felt differently about whether a rigid or flexible routine was best for their child and family. Similarly, there was variability in how caregivers aimed to balance the child’s needs with those of other family members (e.g., parental work schedules, sibling’s bedtime routines). One mother reflected on the need to maintain balance within bedtime routines, as well as all family routines, by saying:

When you have a kid with no condition, ‘cause all kids are unique, right, but…without a diagnosis…you try to have a routine. And you-you try for your kid to understand that routine and why that routine is good for him or for her, but then you have another unique kid that enjoys having routines…each side has, you know…its strengths and its cons, but you just gotta try to keep a balance. I’s not easy to keep a balance even for us that are adults…that’s the key, try to keep a balance. Routines [that] don’t…become an obsession because that’s not good as well. – Participant 28.

Caregivers described using a variety of strategies to meet the child’s sensory needs at bedtime and create a sleep environment they felt helped their child to settle down to sleep. These included activities such as a warm bath, listening to music, putting on white noise, turning on night lights, arranging stuffed animals on/around the child, massage/rubbing back, and layering blankets. Electronics were also used during bedtime routines, often with time limits or content parameters that balanced the child’s preference for using the device with the caregiver’s goal of minimizing stimulation ahead of sleep.

Medication, usually melatonin, was a part of the bedtime routine for some children. At times it was used consistently every night and in other cases, caregivers only used it when their child was displaying difficulty falling asleep. In a few cases, caregivers described that they had previously used medication but had discontinued it because it was ineffective. Thus, for some families, managing medication was yet another domain to balance.

Four caregivers (13.3 %) reported they did not have a specific bedtime routine. These caregivers also reported wide variability in the time the child fell asleep from night to night. One family specifically reported they chose not to have a bedtime routine because they had trialed a routine in the past and it did not help (*“we don’t have a bedtime routine for [him], because it doesn’t help, like, he doesn’t sleep” – Participant 22*).

### Managing night wakings

Management of night wakings was context dependent. Preferences for how to manage night wakings varied across caregivers. When the child’s night waking was disruptive to the caregiver’s sleep, caregivers often used calming strategies similar to those used during the bedtime routine to help the child ease back into sleep quickly and prevent a prolonged episode of being awake during the night.

Sleeping with the child through the night was a strategy employed by 5 caregivers to promote the continuity of the child’s sleep and minimize the disruption of night wakings. Caregivers described that their decision to co-sleep was influenced, partially, by a desire to balance their child’s sleep needs with their own. One caregiver reported that co-sleeping was also their preference and consistent with their family’s cultural norms. However, the four others described that although they wanted to change the current co-sleeping arrangement, they felt co-sleeping helped their child to sleep through the night and they, in turn, experienced better sleep. This decision was not without additional challenges for the family though, as one mother explained:

It’s about 7:45. From then on, I’m indisposed. So, I’m not folding clothes, I’m not doing dishes, I’m not cleaning, like where, when a lot of times that’s what you know, mom dad, whatever are doing, we’re taking me out of the picture for that. I’m also not spending time with my husband…I, we have no alone time, you know, because I’m up there, and normally, mom and dad get alone time when the kids are in bed. We used to, we used to try to watch a show together at night, and just like…just have something that we share. We don’t have that at all…So in terms of okay I’m sleeping a little bit better, but I’m missing out on all of that. – Participant 16.

Strategies caregivers used to manage night wakings also evolved overtime and in response to their child’s development. For example, one family had a goal for the child to develop strategies to ease himself back into sleep independently and therefore changed their approach. However, the mother was unsure if the change in his night waking behavior was due to this new approach or the child getting older. She explained,

So now it doesn’t look like, “Hey, like, let’s talk this through.” It’s like, “… this doesn’t make sense. Go back to bed.”…He understands that these are things that I [child] can deal with, I can self-cope, or I can figure out. I’m just waking up other people because it feels better that way. Right? Um, it-its just a quick fix, and we want him to do the work himself. So, he will go back to bed. Sometimes he’s allowed to journal, read a book. Um, but I think it’s just a habit for him to just come to us, and he still falls into that pattern sometimes. So it’s a lotta times just sayin’—i—we were told by the ABA therapist, “Don’t give it attention.” Right? “Close the door. Bye. We’re not gonna deal with it.” He used to knock on the door for, like, an hour, and we had to just sit there and listen to it and not feed the—we were supposed to cut it off…Um, so I don’t know if that helped along the way, orI don𥀙t know if [he] just grew up. I\\I can’t attribute it to anything. – Participant 19.

Finally, for two families, maintaining safety at night was a primary concern as their child’s behavior (e.g., leaving the home, breaking things) during an unsupervised night waking could be dangerous. The use of monitors, locks, and having the child sleep in the caregiver’s room were used to maintain supervision of the child overnight and be promptly alerted to night wakings.

### Management effort

Caregiver perceptions of the effort devoted to managing sleep existed on a continuum from easy to hard. Perceptions of effort were not consistently congruent with the time devoted to bedtime, as one mother described, “*ya know what? It’s not even that long. It takes maybe 10 to 15 minutes to get him to bed…when you think about it it’s not even that long. Maybe 20 minutes, max It seems to drag out.” (Participant 30*) Instead, various contextual factors influenced the caregivers’ perception of effort, including the consistency of the routine, the child’s behavior at bedtime, the support of a second caregiver during the bedtime routine, and the caregivers’ overall energy level. For one mother, the consistency of the bedtime routine was one strategy to balance the effort of managing sleep with her own mental health. She explained,

I’ve tried to keep it very routine and consistent, just because it makes my life easier. And I know there is an end in sight. Whereas I know in speaking with other caregivers, bedtime [is] a challenge, and they don’t know how long they’ll be laying in the bed. Then they have to sneak out. And whereas I need to know there’s an end in sight, because he wasn’t always in school full time. So, I at least know for myself, by like 8:30, I can go eat a bag of chips, and he’s not there…or I, you know, I can do what I need to do for myself. And that is something I look forward to, so to keep him consistent helps me out mentally. – Participant 23.

In addition to how current efforts to manage sleep were perceived, caregivers also spoke about ‘past effort’ and ‘anticipating the effort’ needed to make a change in sleep behaviors. Caregivers of children with few or no current sleep challenges, spoke about ‘past effort’ as they reflected that even if their child was now sleeping well, it had not always been that way. In addition, caregivers of children with sleep disturbances who wanted to make a change often spoke about ‘anticipating the effort’ and this was a barrier to changing routines. For example, one mother explained,

He’s not a big kid, but he’s nota little kid, so I really want to find ways for him to feel more at ease and comfortable transitioning into bed without somebody there with him. But I know that’s going to be a very long process. – Participant 1.

The majority of the effort described related to managing sleep were centered around establishing routines at bedtime and managing night wakings. However, caregivers also recounted various factors that influenced their child’s sleep, including daytime exercise/physical activity, illness, naps, and hunger. Thus, family management of sleep involved not only establishing bedtime routines, but also efforts related to maintaining daytime routines and balancing daytime activities to promote sleep at night.

## Discussion

This paper reports on the results of a secondary analysis of qualitative data collected fora study on family management of caregiving challenges in the context of raising a school age (4–10y) child with ASD. Sleep disturbances were frequently described by caregivers, and these qualitative descriptions largely converged with quantitative CSHQ scores. The qualitative exploration of how caregivers manage sleep disturbances revealed that family management of sleep is a balancing act as caregivers work to establish routines and manage night wakings.

Sleep disturbances were described by 70 % of caregivers, which is consistent with the existing literature that estimates that 50–80 % of children with ASD experience sleep disturbances (Goldman et al., 2012; Johnson et al., 2012; Krakowiak et al., 2008; Malow et al., 2016; Souders et al., 2009). When examining specific types of sleep disturbances, night wakings (43.3 %), bedtime resistance (30 %), and sleep anxiety (30 %) were common in this sample of children with ASD. A recent qualitative study using focus group methodology to develop a new parent-rating scale for insomnia in children with ASD identified trouble falling asleep, trouble staying asleep, and early morning wakings as common sleep disturbances (Sinha et al., 2023). Few caregivers in our study discussed early morning wakings as problematic. This was surprising given that early morning wakings have been documented in other autistic samples (Hering et al., 1999; Liu et al., 2006; Richdale & Prior, 1995), including with actigraphy (Souders et al., 2009). A possible explanation is because our sample was younger and waking earlier in the morning is expected at younger ages. Further, caregivers may have normalized early morning wakings and adjusted their daytime schedule to adapt to their child’s earlier wake-up time. Indeed, Sinha et al. (2023) found caregivers’ perception of early morning wakings was context dependent and relative to the times family members needed to be awake for school and work.

Regarding night wakings, several parents described night wakings that were not disruptive to the sleep of other family members. This finding of “contented sleeplessness” was first described by Wiggs and Stores (2004) in a study using actigraphy and parental report. They described that some children with sleeplessness during the night remain in bed, do not signal their parents, and may even display overt contentment by giggling or quietly talking to themselves. However, even if night wakings are not disruptive to other family members, the child being awake for an extended period overnight may be problematic because night wakings are associated with disruptive daytime behaviors such as physical aggression, irritability, and hyperactivity (Mazurek & Sohl, 2016). When night wakings are not disruptive to other family members, caregivers may be unaware of the true frequency and duration of the night wakings. This in turn impacts screening and identification of sleep disturbances in the clinical setting. These findings signal the importance on not relying solely on parental report and instead including self-report when developmentally appropriate and employing objective measures such as actigraphy.

In all cases where the caregiver described challenges with sleep and/or bedtime routines in the qualitative interview, the CSHQ score was also above the cut-off that indicates a clinically significant sleep difficulty. In other words, when the caregiver perceives that there are problems with the child’s sleep, the CSHQ score is also interpreted as indicating a clinically significant sleep concern. In six cases (20 %), the caregiver did not describe any sleep disturbances during the interview, but the CSHQ score was above the cut off (range 42–48). This is consistent with prior work by Johnson et al. (2012) that found caregivers may not endorse a sleep disturbance (evaluated as yes/no to a single question) despite a high CSHQ or Modified Simonds & Parraga Sleep Questionnaire. Our work extends this finding by using the content of in-depth interviews rather than a single question to determine if the caregiver perceives problems with sleep and/or bedtime. This divergence between the caregiver’s perception of the child’s sleep and CSHQ score could indicate normalization of the child’s sleep disturbance. Halliwell et al. (2021) found that families of young adults with ASD experienced ongoing sleep disturbances and often felt resigned to the fact that these sleep disturbances would never resolve. Thus, the young adult’s disrupted sleep as well as the disrupted sleep of caregivers became “normal” for families. This normalization by caregivers could lead to under reporting of sleep disturbances during clinical interviews, especially if a standardized screening instrument is not used. This finding reinforces the critical need to use validated proxy and self-report sleep questionnaires in addition to sleep interviews during routine clinical care.

Caregivers used a variety of strategies to manage bedtime and sleep disturbances in this study. The majority of families had established reliable and predictable bedtime routines, which is consistent with clinical recommendations (Galion et al., 2023; Malow et al., 2012; Vriend et al., 2011) and prior research (Sinha et al., 2023). However, while 86.7 % of caregivers in this study had developed a bedtime routine that aimed to address bedtime struggles and sleep anxiety, it appears bedtime routines alone were not sufficient to establish easy sleep initiation for some of our families. Instead, family management of sleep was a balancing act in which families balanced multiple domains to support their child’s sleep. For some families, routines and management strategies were child-focused while for others the routines and strategies were more family-focused. When routines and strategies were child-focused the caregiver’s efforts were devoted to supporting the child’s sleep needs and less consideration was given to the caregiver’s own sleep needs. Routines tended to be more rigid and effortful for the caregiver. In contrast, family-focused routines had increased flexibility to accommodate the needs of other family members (e.g., sibling’s sleep schedule, parental work schedule) and promoted the child’s independence in carrying out the routine. Additional research is needed to evaluate the child, caregiver, and family factors that contribute to routines being more child- or family- focused. Research on how child- or family- focused bedtime routines impact child and family outcomes (e.g., sleep duration, sleep quality, family quality of life) is also needed.

A notable area of divergence in our study from clinical recommendations was the use of electronics at bedtime. In research, use of electronics and screen-time before bed have been associated with poorer sleep and more disruptive daytime behaviors (Dong et al., 2023; Engelhardt et al., 2013). However, caregivers in our study that incorporated electronics (e.g., tablet, TV) into the bedtime routine did so because, in their experience, this strategy helped the child to settle down. The impacts of electronics use on sleep include time displacement, increased psychological stimulation, and light from the device influencing melatonin release and circadian timing (LeBourgeois et al., 2017). Caregivers often used time limits or restricted the type of media being viewed, which serves to reduce some of the potential negative impacts of electronics use on sleep. However, these strategies may not fully mitigate the impact of light exposure from the electronic device. Families can benefit from help with negotiation strategies that allow their children can have screen time until the established bedtime routine start time. This negotiation should include replacement of screen-time with caregiver 1:1 time that includes calming activities (e.g., yoga together as a family, massage, Lego building, family prayer) for 20–30 min prior to bedtime. These calming activities for 20–30 min before bedtime should done even if child falls asleep easily at bedtime. Night waking is the biggest concern in this study and calming activities can be done in the middle of the night when the child wakes up by themselves. If the routine is practiced every night with the child, they are more likely to self soothe with these activities when they wake up in the night (Souders et al., 2017).

### Implications for nursing practice & research

In the clinical pathways for treating early and middle insomnia (Galion et al., 2023; Malow et al., 2012), screening annually for sleep disturbances and supporting the implementation of behavioral and environmental approaches are two areas in which nurses are well-positioned to support families of children with ASD. Screening for sleep disturbances in clinical practice most commonly occurs via a clinical interview or questionnaire due to this method being low cost, time efficient, and a low burden for families (Moore et al., 2017). However, due to the complexity of sleep disturbances in children with ASD, a multi-method assessment of sleep that includes the above subjective assessments by parents as well as self-report and objective measures (e.g., actigraphy (ActiWatch, Fit Bit, Apple Watch) and polysomnography) may be beneficial. For example, actigraphy is a method of measuring movement via an accelerometer in a device worn on the wrist, ankle, waist, or chest and can be used to estimate periods of sleep and wakefulness (Smith et al., 2018). Our study identified that children with ASD often had night wakings that were non-disruptive to family members, which could impact caregivers’ ability to report on the frequency and duration of these night wakings. Use of actigraphy as a part of a multi-method sleep assessment has the potential to precisely define the severity of night wakings among children with ASD. However, additional research is also needed to ensure that actigraphy algorithms are accurately distinguishing night wakings from other limb movements during sleep in this population (Alder et al., 2020). Wrist-worn actigraphy may also be difficult for some children with sensory sensitivities to tolerate. One strategy to mitigate this limitation is to place the actigraphy device ina pajama shirt pocket (Adkins et al., 2012; Souders et al., 2009; Souders et al., 2017), though this needs to be customized to each individual.

To date, the most comprehensive sleep care has been documented and disseminated by Mallow and colleagues (2012) and the Autism Care Network. The Autism Care Network has created and published 23 tool kits for families to help their children, adolescents, and adults with problems that occur frequently with ASD. Three toolkits address sleep with foci on strategies for children, strategies for teens, and melatonin use. These toolkits are free and available via the internet (see: https://autismcarenetwork.org/toolkits/). Nurses can use these toolkits to provide education about developmentally appropriate sleep habits, discuss sleep issues that are worrisome, and help families to accommodate their child’s unique developmental, behavioral, and sensory needs while balancing the sleep needs of other family members (See Table 4). Establishing positive bedtime routines are recommended for all children to promote healthy sleep hygiene. The results of this study suggest that the added complexity of navigating rigid routines, accommodating sensory needs, balancing the needs of family members, and managing daytime behaviors can create unique challenges for caregivers of children with ASD. In addition, making desired changes to sleep routines, such as co-sleeping, can be inhibited by caregiver perceptions of the effort that it will require. Thus, not only the child, but also the caregiver and family need to be considered when providing sleep education.

### Limitations

While this study provides useful insight into how caregivers of children with ASD experience and manage their child’s sleep disturbances, results should be interpreted in light of several limitations. This study was a secondary qualitative analysis and although questions about sleep were included in the interview guide, it was not the primary focus of that study. Further, caregivers in that study were recruited without inclusion criteria related to their child having sleep disturbances. This makes our findings regarding the high prevalence of sleep disturbances more generalizable. Future research may consider purposive sampling to include caregivers of children with different types of sleep disorders, such as obstructive sleep apnea or restless leg syndrome, and varying severity to expand upon our findings. Finally, caregivers were recruited via a large academic medical center that provides specialty care to children with ASD and there was limited sociodemographic diversity among the respondents. Further research in a more diverse sample is needed.

## Conclusion

The prevalence of sleep disturbances remains very high in the ASD population with approximately 70 % of families identifying a sleep disturbance in their children with ASD. Caregivers of children with ASD expend considerable effort to balance multiple domains (e.g., the child’s sleep needs, the sleep needs of the caregiver and other family members, the child’s sleep environment preferences, daytime activities that promote or disrupt sleep) to promote their child’s sleep, but sleep disturbances remain highly prevalent. Nurses can play an integral role in screening for sleep disturbances, educating families about sleep health, and providing guidance for implementing behavioral and environmental interventions. It is critical for nurses to be trained in the evidence-based behavioral and environmental strategies that improve sleep and develop skills to tailor the treatment for each family’s specific needs and preferences. Future research using implementation science should focus on training nurses to interface with families and tailoring behavioral and environmental sleep interventions across care settings.

## Figures and Tables

**Table 1:** Sample Characteristics.

Characteristic	Caregivers		Children	
	(n = 30)		(*n* = 30)	
	n	%	n	%
**Gender**				
Female	27	90	10	33.3
Male	3	10	20	66.7
**Ethnicity**				
Hispanic	4	13.3	6	20
Non-Hispanic	26	86.7	24	80
**Race** ^a^				
American Indian or Alaska Native	1	3.3	1	3.3
Asian	4	13.3	4	13.3
Black or African American	4	13.3	5	16.7
Native Hawaiian or Other Pacific Islander	1	3.3	1	3.3
White	22	73.3	24	80
Prefer Not to Answer	1	3.3	1	3.3
**Education**				
High School Diploma	8	26.7		
College Degree	12	40		
Graduate Degree	8	26.7		
Other	2	6.7		
**Employment**				
Student or Trainee	2	6.7		
Working Less Than 35 Hours/Week	5	16.7		
Working 35 Hours/Week or More	16	53.3		
Unemployed	2	6.7		
Unable to Work Due to Illness or Disability	3	10		
Unable to Work due to Child’s Illness or Disability	2	6.7		

a Multiple race categories could be selected.

**Table 2:** Sleep Disturbances: Categories, Codes, and Exemplar Quotes.

Category	Codes	Exemplar Quotations
Bedtime	Difficulty Settling Down	It takes a while to get him settled down and ready to sleep because he just wants to keep watching, keep doing. (*Participant 1*)
Resistance	Making Requests Coming Out of the Room	Every night, it’s like “I need my- I need my ‘this’ I need my ‘that’. I need my blankets. I need my friends. I need like ‘this’ in my bed”…He’s trying to get me to not leave the room. (*Participant 30*) He’ll come out between like 8:30 and probably 10 o’clock. They’ll come out a few times, and I’ll be like “back in your room”, and we’ll walk ‘em back in there. (*Participant 15*)
Sleep Anxiety	Needing Parent to Fall Asleep Needing Parent Through the Night[IUSP] Specific Fears	He has to fall asleep ‘cause if my husband tries to get out of bed, he’ll get out of bed. (Participant 27) I’m up ‘til you know 10, 11, 12, you know, whenever and I’m sitting there next to him, and I see how often he reaches for me. (*Participant 16*) Intense anxiety about going to sleep. Like I said, very unreal, um, not- not logical anxiety. Um, snakes coming up to get him. And it was all surrounded around sleep. (*Participant 19*)
Night Waking	Independent Night Waking Night Waking with Parental Involvement Coming into Parent’;s Bed	Sometimes we hear him at 4 am. Or 5 am. For a little bit like talking to himself. Um, and then he goes back to sleep. So, the way that I know that sometimes he’s awake is like stuff is moved around in his room. (*Participant 2*) Um, if I’m there she might go back to sleep or she might go back to sleep in an hour. (*Participant 5*) I don’t even know she’s there ‘til I wake up in the morning. Like, she doesn’t wake me up. She just climbs in the bed and goes to sleep. (*Participant 14*)
Other	Restless Sleep Nightmares or Night Terrors Daytime Sleepiness Early Morning Rising Obstructive Sleep Apnea	She tosses and turns. (Participant 29) She wakes up crying a lot or having nightmares or talking in her sleep, usually about things that are, like, bothering her. (*Participant 29*) 1:30p to 3:15p that he’s in class is a really hard time for him to stay awake. He typically will sleep through that if he can. (*Participant 21*) A typical day, [he] wakes up before the crack of dawn. We were having a hard time where he was waking up at 3:00–4:00 a.m. and wouldn’t go back to sleep. (*Participant 8*) He has obstructive sleep apnea. He has had his tonsils and his adenoids out because he—I mean, it was bad, really bad when he was little.He was like a freight train snoring.(*Participant 21*)

**Table 3:** Family Management of Sleep: Sub-Themes, Codes, and Exemplar Quotes.

Sub-Theme	Codes	Exemplar Quotes
Establishing Routines	Consistent, Predictable Routine Inconsistent Routine Parent Leads Bedtime Routine Child Leads Bedtime Routine Rigid Routines Flexible Routines Electronics during Bedtime Routine Sensory Needs at Bedtime Managing Medication	He generally just enjoys winding down, and getting in his pajamas, putting on his jazz playlist. I think ‘cause it’s a routine, and we’ve made it a positive routine. (*Participant 11*) I wanna say it’s almost non- almost nonexistent because…it’s a, I feel like it’s different every day. (*Participant 13*) I usually give him a lot of different warnings about how soon bedtime is going to be and when it’s time (*Participant 1*) Sometimes I’m- I’m distracted doing stuff, and she’s like, mom, it’s almost time. I gotta go, so goodnight. (*Participant 28*) he’s not easy-going, so it- it has to be the routine (*Participant 27*) Realizing, you know, not every night at the exact same time are they gonna wanna do the exact same thing. But, you know, “Okay, 7:20. Okay, now you can come downstairs. You get, you got an extra five minutes tonight. Let’s now calm down. Get ready to go to bed.” And you can make small adjustments here and there. (*Participant 10*) I put a timer on the TV so that they don’t – he’s not too stimulated. You know, I- I don’t mind him watching TV ‘cause it’s not like he’s watching anything bad. (*Participant 4*) He makes me pile every one of his toys on- every one of his stuffed animals on top of him. They keep him safe, he says. He needs his friends. He has one, two, three nightlights and sound machines in his room, that I have to turn on. (*Participant 30*) [She] has to have at least 20 to 30 min of time before we start the bedtime routine. She has to have her melatonin 20 to 30 min before that or else she’s not in the window where she can fall asleep comfortably. (*Participant 20*)
Managing Night Wakings	Actively Supporting the Child Back to Sleep Staying With the Child Through the Night Planned Ignoring Maintaining Safety	Rub her back; that’s her go-to to calm down. Like, I do, like, a little rub-and-scratchin’ thing on her back…And it usually relaxes her. And then, um, I have to hold her and make sure—again, hug and squeeze. She likes tight squeezes. And sometimes it’s just a matter of her just easin’ back into sleep. (*Participant 29*) He doesn’t wake up throughout the night as much as he used to, but he stirs a lot which is part of why I’ll often be reluctant to actually get up and leave the room. Because I don’t, you know, I don’t want to have to go back again in five minutes because he stirred. (*Participant 1*) So, I don’t get up and rush to him. Where ifhe doesn’t call me again, I won’t get up. You know? I—I know nothing is wrong, but he might say, “Oh. I had a nightmare.” And I think he just, kind of, woke up turning over and he considers it a nightmare (*Participant 8*) We keep his door locked on his room, so I mean, unfortunately, that means that he still wears an overnight diaper, and you know he’s gonna be nine years old, and he’s bigger, and you know I hate that, that might be something he has to do forever. I would love for him to get to the point where he would be able to get up in the middle of the night and use the bathroom…at this point, that would not be safe for him to do independently (*Participant 2*)
Management Effort	Perception of Effort – Easy Perception of Effort – Hard Past Effort Anticipating Effort Balancing Daytime Activities to Promote Sleep	Going to bed is easy. We have like a set routine that he’s known for like a very, since I don’t know, forever. (*Participant 25*) bedtime is like, if I have to say, that is the most challenging for us…personally, because it’s at the end of the day. Just want the day to end, we’ve been through the day, our energies are at the lowest level. So just want her to go to bed soon. (*Participant 5*) There were times where I would literally have to sit on the floor outside the bedroom door to get him to just stay in the room ‘cause I would have to catch him as he came out of the room. (*Participant 9*) They’re sleeping in the living room, which, you know, maybe is not the greatest, but, again, a battle I don’t wanna fight. (*Participant 9*) Definitely the amount of stimulation throughout the day has a big effect. Um, if he hasn’t been able to move his body enough, he’ll often be really restless and cranky going to, trying to lay down in bed. Um, but if we um had a particularly long, eventful day and he goes to sleep too late then that is a big trigger for night terrors. So yeah, again, it’s a big (emphasis) balancing act. (*Participant 1*)

**Table 4:** Behavioral and Environmental Strategies to Improve Sleep.

Strategy	Rationale	Tips to Help Families Implement
Exposure to bright natural light first thing in the morning	The sun helps to set the body’s circadian rhythm and internal clock. Exposure to bright natural light signals to the body that it’s time to be awake and alert.	Open blinds to let in morning light. Brainstorm with the family other ways to incorporate early morning sun into the family’s routine. Consider getting to the bus stop early, taking a short morning walk, or sitting outside for breakfast.
Exercise for 30 min during the morning or early afternoon	During exercise, muscles secrete hormones that regulate the body’s circadian rhythm and promote relaxation.	Talk with families about their child’s interests to find an exercise activity that is enjoyable. The child may need to start slowly and build up to 30 min. Some exercise ideas are: dancing, yoga, swimming, jumping on a trampoline, throwing a medicine ball, or building an obstacle course.
Eat a balanced diet	The timing of meals can impact the body’s circadian rhythm, and a balanced diet can improve sleep.	Space meals throughout the day and try to keep that same meal timings on weekdays & weekends. Consider serving a lighter dinner to prevent the child from feeling “too full” at bedtime. Avoid foods and drinks with caffeine (e.g., chocolate, soda, coffee, tea) and high sugar content in the late afternoon and evening.
Help your child to have a great day	Anxious thoughts and feelings throughout the day can lead to an anxious evening. This can make bedtime routines and falling asleep more difficult.	Work with the school to ensure appropriate educational supports. Maintain open communication with the teachers and therapists and inquire about daytime behavior, including sleepiness. Monitor the sensory environment and reduce sensory inputs that are too stimulating (e.g., use noise cancelling headphones). Take a break after school to wind down and reset the “social battery”
Create a calming evening environment	Reducing light in the evening helps with regulating the body’s circadian rhythm.	Dim lights down about 2 h before bedtime (i.e., “romantic” lighting). Opt for red, orange, or yellow light in the evening hours or if using a nightlight. Aim to turn off devices (e.g., iPads, TV, Computer, iPhone) about 45–60 min before bedtime. If using devices close to bedtime (e.g., for communication), avoid blue light by using evening screen options. Decrease environmental commotion and reduce noise throughout the home.
Create a visual schedule for the bedtime routine	A visual schedule helps to create a bedtime routine that is clear and predictable. This can reduce anxiety and promote independence with going to bed.	Develop a bedtime routine that starts about 20–30 min before bedtime and does not include any technologies with artificial light. Write out the steps and include pictures or cartoons to provide visual supports. Create the visual schedule together as a family to promote the child’s engagement and inclusion of siblings with the routine.
Engage in relaxing activities before bedtime	Calming activities can activate the parasympathetic system and improve sleep.	Include 1 or 2 relaxing activities in the bedtime routine. These can be activities for the whole family or 1:1 with the child. Some ideas are: puzzles, Legos, building objects, coloring, yoga, deep breathing exercises, massage, warm bath, rocking chair, reading. For some children, brushing teeth right before bedtime can be alerting. Consider a trial of putting teeth brushing right after dinner (if doing so, offer only water until bedtime).
Create a cool, dark, quiet, and boring bedroom	Darkening and cooling when the sun sets are physiologic cues for sleep.	Use a fan to create a cool environment. Black out curtains can help to darken the room and add a small nightlight with red, orange, or yellow light if the child has a fear of the dark. A quiet room can include a white or brown noise machine. To create a boring environment, remove clutter and consider storing toys outside of the bedroom. Putting the bed against the wall also helps to promote sleep via a feeling of nesting.
Pay attention to sensory cues for sleep	Sensory cues or “zeitgebers” help to set the circadian rhythm and cue the body that it is time to sleep.	The bedroom should “feel like sleep, look like sleep, smell like sleep, and sound like sleep”. Examples of sensory cues include: cozy pajamas, a heavy quilt or sleeping bag, a scented plug-in (e.g., lavender), a white or brown noise machine.
Consider Location of Routine	Stimulus control is a hallmark of cognitive behavioral therapy for insomnia. Going into the bedroom when “irresistibly sleepy” helps to associate the bedroom with sleep.	For children with difficulty falling asleep, it can be helpful to do most of the routine outside of the bedroom and then go into the bedroom as the last step when “irresistibly sleepy”. This helps to associate the sensory cues of the bedroom with feelings of sleepiness.
